# Osteoid osteoma (OO) of the coracoid: a case report of arthroscopic excision and review of literature

**DOI:** 10.1051/sicotj/2015016

**Published:** 2015-07-10

**Authors:** Saumitra Goyal, Hatem Galal Said

**Affiliations:** 1 Assiut Arthroscopy & Sports Injuries Unit, Orthopaedic & Traumatology Department, Assiut University Assiut 71515 Egypt

**Keywords:** Osteoid osteoma, Shoulder, Coracoid, Arthroscopic excision, Technique

## Abstract

Osteoid osteoma (OO) of the coracoid is a rare entity that may present with variable symptoms from shoulder leading to delay in diagnosis and treatment. We present the clinical and radiological findings and management of one such case along with a review of similar cases reported in the literature. There was a delay of 2 years in diagnosis, which was later confirmed by computed tomography in addition to magnetic resonance imaging (MRI). The lesion was accessed arthroscopically and excised by unroofing and curettage. “OO” should be included in the differential diagnosis of shoulder pain in young patients not responding to long-term conservative treatment. Arthroscopic excision and curettage provide a good choice for management, with low morbidity and rapid recovery.

## Introduction

Following the first description by Jaffe in 1935, osteoid osteoma (OO) is most commonly seen as benign bone forming lesion in the diaphysis of long bones [1, 2]. It rarely occurs in flat bones and the involvement of scapula is reported to be about 1% among all cases of “OO” [3]. The involvement of the coracoid process has been reported sporadically in the literature [4–18]. These cases presented with shoulder pain and were treated for long durations for nonspecific diagnoses ranging from cervical radiculopathy to “periarthritis” leading to delay in final diagnosis [4–18]. From more extensive surgical excision to percutaneous radiofrequency ablation, many treatment modalities have been described for such lesions with good outcomes [4–18]. We present here a case report of a patient who had delayed diagnosis of coracoid “OO” and eventual excision of the lesion by arthroscopic technique, along with a review of similar cases from the literature.

## Case report

A 27-year-old manual worker presented with a complaint of left shoulder pain for two years. The pain was insidious in onset, gradually increasing and worse at night and with activity eventually affecting his daily work. He gave a history of occasional radiation of pain to the lateral aspect of his arm and up to the elbow. There was no history of trauma, dislocations or weakness of the shoulder. The pain was partially relieved by the use of anti-inflammatory medications. There was no history of any substance abuse or any co-morbid illness. He reported to have undergone a shoulder diagnostic arthroscopy without any improvement; intra-operative details were unavailable. Physical examination did not reveal any local signs of inflammation except for mild tenderness over the bicipital groove. He had a full range of flexion (0–180), abduction (0–160), external (0–80) and internal rotation (up to T12) without signs of impingement or instability. Mild wasting of muscles was noted around the shoulder but neurological examination was normal. Mild scapular dyskinesia was also noted on the left side.

**Figure 1.**
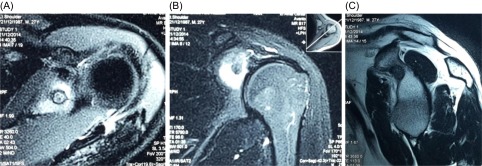
(A and B) T2-weighted MRI images showing high signal intensity within the glenoid around the lesion with low signal, (C) T1-weighted sagittal image reveals a low signal lesion at the base of the corocoid.

**Figure 2.**
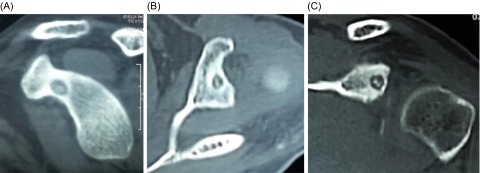
(A–C) CT scan in sagittal, transverse and coronal planes reveals the typical lesion of osteoid osteoma (OO) with a central nidus and surrounding sclerotic bone (to localize lesion pre-operative and plan excision).

Initial radiographs and inflammatory markers did not reveal any abnormality. He was advised to undergo an MRI of the left shoulder and cervical spine. The T2-weighted MR images (Figures 1A and 1B) revealed a circular lesion with a rim; the “target lesion” with central low intensity surrounded by hyperintense signal in the surrounding bone and the T1-weighted sagittal plane image (Figure 1C) revealed a well-defined nodular lesion of low signal intensity at the base of the coracoid process. The CT scan (Figure 2) confirmed a 7 mm “nidus” of “OO” at the base of coracoid process; within the glenoid notch. The MRI of cervical spine was normal. Considering the symptoms along with radiological confirmation of “OO”, the patient was planned for arthroscopic excision of the lesion.

**Figure 3.**
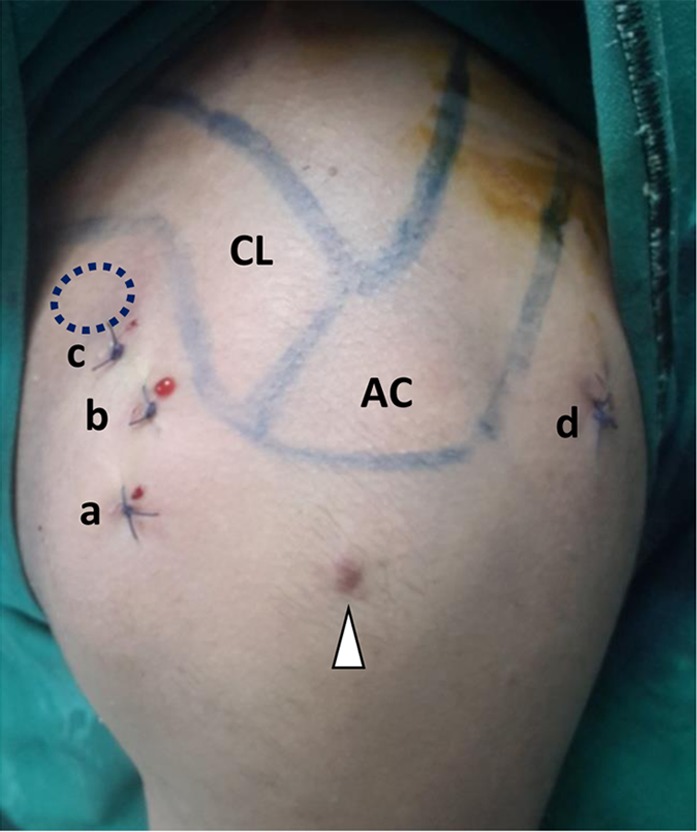
Postoperative image showing portals used for arthroscopic approach to the base of coracoid (a and b) standard anterior portal, (c) additional anterior portal just lateral to coracoid process, (d) standard posterior portal, CL – clavicle, AC – acromion, arrowhead – previous arthroscopy scar, dotted circle – coracoid process

**Figure 4.**
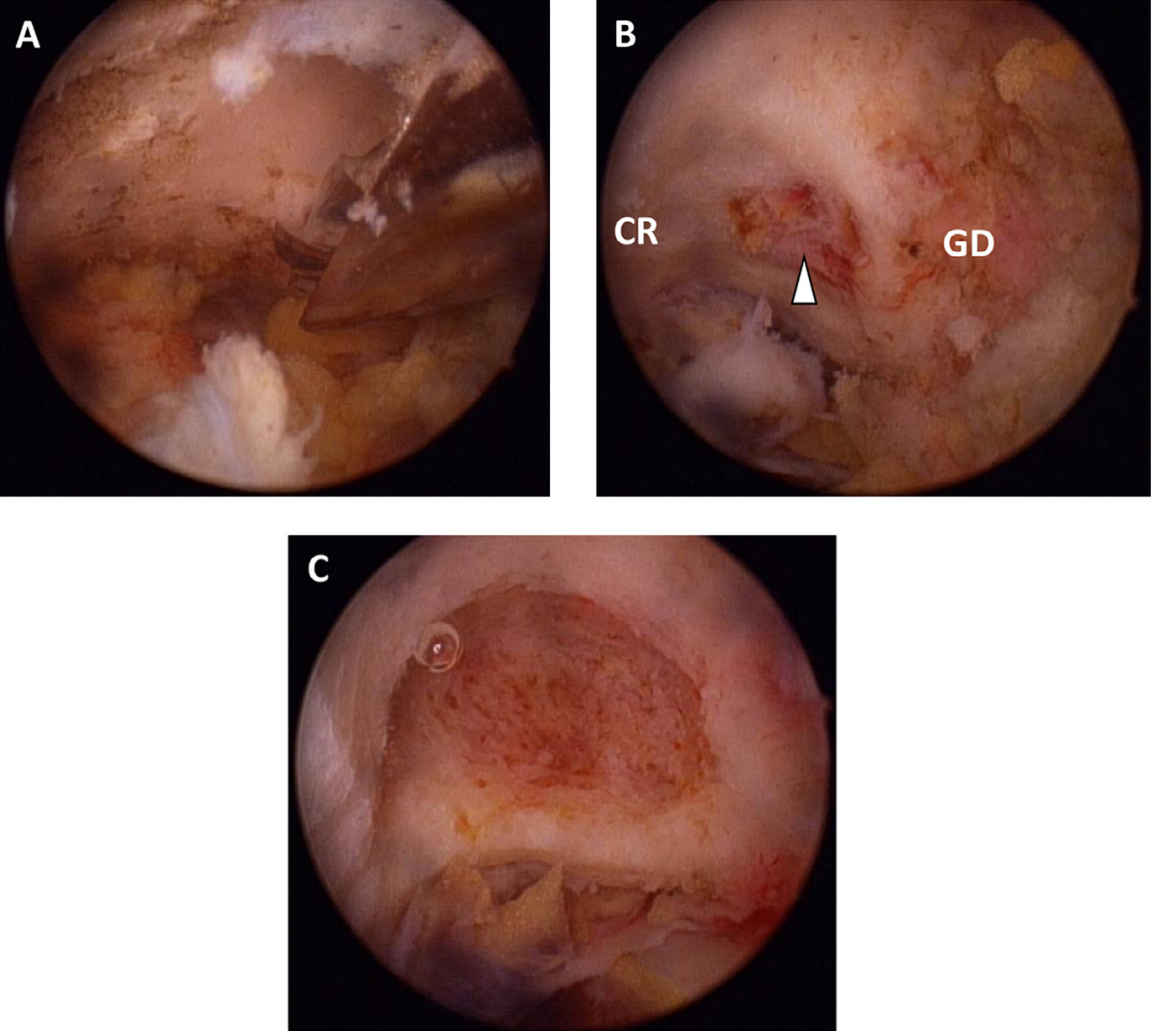
Arthroscopic images of excision of osteoma. (A) De-roofing of the lesion by arthroscopic burr, (B) after removal of anterior wall revealing the hypervascular nidus (arrowhead) at the base of coracoid (CR) medial to the glenoid (GD), (C) after complete removal of lesion there is healthy cancellous bone at the base.

We performed diagnostic shoulder arthroscopy to rule out any intra-articular pathology using standard posterior and anterior portals in the beach chair position. An additional anterior portal was created under vision just lateral to the coracoid (Figure 3). The rotator interval was cleared (Video 1) and irregularity of bone at the neck of glenoid was noted. This area of irregular bone was excised by burr and de-roofed to expose nidus of the lesion (Figure 4A, Video 2a and 2b). The typical fibrous nidus rich in vacular tissue was revealed in the centre (Figure 4B) and the material was taken for histopathology. The lesion was curetted till the sclerotic margin was cleared and healthy bone was visible (Figure 4C, Video 3). No other procedure was performed.

The patient reported resolution of pain immediately following surgery and was permitted to carry out normal activities. The biopsy confirmed “OO” and at the last follow-up at three months the patient showed complete resolution of symptoms.

## Literature review

Kaempffe published the first case report of osteoid osteoma of the coracoid [4]. Since then about 15 more cases have been reported, with most reports initially missing the treatment of osteoid osteoma [4–18]. The cases reported thus far are between 12 and 34 years of age with 13 males and 3 females with a heterogeneous mix of presentation (Table 1). Although, the consistent presenting feature was shoulder pain which worsened at night and was relieved by NSAIDs additional features like radiation of pain and restriction in shoulder movement led to misdiagnoses of these cases as impingement syndrome, adhesive capsulitis, synovitis, arthritis or cervical radiculopathy even though none reported positive neurological signs [4, 7, 9–13, 15, 18]. Most patients received nonspecific treatment in the form of analgesics, physiotherapy, intra-articular steroid injections, etc., and had a delay in diagnosis ranging from two months to four years (mean of 16.3 months) [4–18]. Angius et al. in 2007 and Ghosh et al. in 2011 reported between them three cases of osetoid osteoma of the scapula; close to the coracoid and spinoglenoid notch presenting as brachial plexopathy and neurologic amyotrophy further complicating the diagnosis [19, 20]. Initial radiological investigation was limited to X-ray with magnetic resonance imaging (MRI) in a few cases. Eventually they underwent definitive treatment for osteoid osteoma after further imaging in the form of MRI, CT scan and Tc99 scintigraphy was done (Table 1). The majority of the cases (12 out of 16) underwent open surgical excision and the rest were treated with minimal invasive techniques like percutaneous radiofrequency ablation, or CT guided biopsy (Table 1). Kelly treated one case successfully through arthroscopy [8].

**Table 1.** Summary of cases reported in the literature [4–18].

Author(s) *Journal*, *Year*	Case profile	Initial diagnosis; treatment	Confirmatory investigations	Final treatment
Rouhani A et al. *ABJS*, *2014*	25 year male left shoulder chronic pain (27 months)	Cervical discopathy/impingement syndrome	MRI, CT, Tc99 bone scan	Surgical excision – deltopectoral approach
Gharahdaghi M et al. *Iran Red Cres Med J*, *2013*	12 year male right shoulder chronic pain (9 months)	Nonspecific	Tc99 bone scan, CT scan	En bloc resection of 2 cm segment of coracoid – mini deltopectoral approach and screw fixation
Gogoi P et al. *Sci J Clin Med*, *2013*	12 year male right shoulder chronic pain (4 months)	Nonspecific	CT scan (4 mm nidus)	Surgical excision – deltopectoral approach
Pourfeizi HH et al. *MJIRI*, *2012*	34 year female right shoulder chronic pain (4 years)	Cervical radiculopathy	Scintigraphy, CT scan (1 cm nidus)	Surgical excision – Roberts approach with coracoid osteotomy and screw fixation
Mavrogenis AF et al. *J Sho Elb Surg*, *2012*	12 year Male Chronic pain (8 months)	NA	NA	Surgical excision
Glanzmann MC et al *J Sho Elb Surg*, *2011*	22 year male pain and restriction of movement (18 months)	Adhesive capsulitis; unsuccessful arthroscopy	MRI, CT scan	Arthroscopy guided drilling and thermo-ablation
Lee BG et al. *J Orthop Sci*, *2010*	21 year male right shoulder chronic pain (16 months)	Synovitis with suspected chronic osteomeylitis; arthroscopic synovectomy and antibiotics	MRI, CT scan repeated at 5 months (delayed appearance of nidus)	Surgical excision – deltopectoral approach, entire coracoid excised
Kossmann N et al. *Praxis*, *2010*	21 year male right shoulder chronic pain (10 months)	Nonspecific	CT scan (missed in initial MRI)	CT guided excision
Ishikawa Y et al. *J Sho Elb Surg*, *2005*	17 year female left shoulder pain and restricted movement (2 months)	Synovitis of shoulder with osteomyelitis scapula	CT scan (6 mm nidus), missed in initial MRI	Surgical excision – deltopectoral approach
Marquardt B et al. *J Shou Elb Surg*, *2005*	21 year male right shoulder chronic pain (1 year)	Nonspecific symptomatic, then misdiagnosed as soft tissue sarcoma	Tc99 scan, MRI, CT scan	CT guided percutaneous radio frequency ablation
Kelly AM et al. *Arthroscopy*, *2002*	12 year male right shoulder chronic pain (6 months)	Nonspecific	MRI (5 mm nidus)	Arthroscopic excision
Akpinar S et al. *Bull Hops Jt Dis*, *2001*	14 year female right shoulder chronic pain (4 years)	Arthritis	MRI, CT, Tc99 bone scan	Surgical excision – deltopectoral approach with coracoid osteotomy and screw fixation
Gracia IA et al. *J South Ortho Assoc*, *2001*	18 year male right shoulder chronic pain (2 years)	Nonspecific	MRI	Surgical excision – anterior approach
Ogose A et al. *CORR*, *1999*	*Two cases* 14 year male left shoulder chronic pain (1 year)	Intraarticular steroid injection	Radiograph	Curettage – repeated at 1 year
	17 year male right shoulder chronic pain (4 months)		CT scan	Surgical resection (approach not specified)
Kaempffe FA. *CORR*, *1994*	14 year male right shoulder chronic pain (14 months)	Nonspecific	Tc99 bone scan, CT scan	Surgical excision – Swafford and Lichtman posterior approach

## Discussion

Osteoid osteoma of the coracoid is a rare entity, hence it is not considered commonly in the differential diagnosis for chronic shoulder pain in young adults [3, 5]. Juxta-articular osteomas have unusual symptoms and presentation which make diagnosis difficult and delayed by up to 26.6 months in comparison to 8.5 months in such cases [21, 22]. Shoulder pain with associated features of pain radiation and presence of muscle atrophy, which is probably secondary to disuse, or night pain with restricted rotation often leads to these patients being misdiagnosed and wrongly treated as having cervical radiculopathy and “periarthritis”.

There is a delay in diagnosis because of inadequate early radiological evaluation. The importance of this aspect was reported by Ogose et al. who suggested that either CT scan or MRI should be done early if there is suspicion of osteoid osteoma. These investigations reveal features that point to a definitive diagnosis of osteoid osteoma although sometimes there may be a late appearance of the nidus [5, 12]. Since MRI is the more frequent radiological investigation performed for shoulder pathologies, the presence of high signal intensity on T2-weighted images with or without definite “nidus” should raise the suspicion of osteoid osteoma.

Percutaneous radio frequency ablation (RFA) is now the treatment of choice for osteoid osteomas [23]. Most patients though have undergone open surgical excision for the cases of coracoid “OO” reported in the past (Table 1). Surgical excision requires a thorough dissection through a deltopectoral approach to properly visualize the nidus which can be associated with a significant morbidity. A percutaneous procedure may pose a risk to neurovascular structures running close to the base of coracoid and may also be limited by the availability at all centres.

Arthroscopic approach to the base of coracoid as described by us allows direct visualization of the lesion as opposed to percutaneous methods. Also it has less morbidity compared to open surgical excision and en bloc resection. The arthroscopic method is appropriate for the excision and curettage of osteoma around joints like hip and shoulder by direct visual confirmation [8, 24].

## Conclusion

Young adults with chronic shoulder pain especially with night pain with nonspecific diagnosis should be evaluated early with MRI or CT scan to rule out a bone lesion. The presence of high signal intensity on T2-weighted MR images should raise suspicion of “OO”. Coracoid lesions may be even more difficult to diagnose due to variable shoulder symptoms but localization by early advanced imaging would allow successful treatment by arthroscopic de-roofing and curettage with low surgical morbidity and avoiding risk to neuro-vascular structures close to coracoid process.

## Online material

**Video 1** – Release of the rotator interval and exposure of the base of coracoid **Video 2a** – Removal of the roof using arthroscopic burr to access the nidus. **Video 2b** – Removal of the roof using arthroscopic burr to access the nidus. **Video 3** – Curettage and extraction of the hypervascular tissue of the osteoid osteoma

## Conflict of interest

SG and HGS declare no conflict of interest in relation with this paper.
